# An Aurora kinase inhibitor, AMG900, inhibits glioblastoma cell proliferation by disrupting mitotic progression

**DOI:** 10.1002/cam4.1771

**Published:** 2018-09-17

**Authors:** Jaewook Ryu, Jaehyuk Pyo, Chang‐Woo Lee, Ja‐Eun Kim

**Affiliations:** ^1^ Department of Biomedical Science Graduate School Kyung Hee University Seoul Korea; ^2^ Department of Molecular Cell Biology School of Medicine Sungkyunkwan University Suwon Korea; ^3^ Department of Pharmacology School of Medicine Kyung Hee University Seoul Korea

**Keywords:** AMG900, anti‐cancer drug, Aurora kinase, glioblastoma, mitosis

## Abstract

The Aurora kinase family of serine/threonine protein kinases comprises Aurora A, B, and C and plays an important role in mitotic progression. Several inhibitors of Aurora kinase have been developed as anti‐cancer therapeutics. Here, we examined the effects of a pan‐Aurora kinase inhibitor, AMG900, against glioblastoma cells. AMG900 inhibited proliferation of A172, U‐87MG, and U‐118MG glioblastoma cells by upregulating p53 and p21 and subsequently inducing cell cycle arrest and senescence. Abnormal cell cycle progression was triggered by dysregulated mitosis. Mitosis was prolonged due to a defect in mitotic spindle assembly. Despite the presence of an unattached kinetochore, BubR1, a component of the spindle assembly checkpoint, was not recruited. In addition, Aurora B was not recruited to central spindle at anaphase. Abnormal mitotic progression resulted in accumulation of multinuclei and micronuclei, a type of chromosome missegregation, and ultimately inhibited cell survival. Therefore, the data suggest that AMG900‐mediated inhibition of Aurora kinase is a potential anti‐cancer therapy for glioblastoma.

## 1 INTRODUCTION

Aurora kinases are serine/threonine kinases responsible for faithful mitotic progression. The Aurora family comprises Aurora A, B, and C, which share a C‐terminal catalytic domain but possess different N‐terminal regulatory domains.^1^ These kinases show both distinct and overlapping characteristics with respect to localization and functions. The subcellular localization of the different kinases is dynamic throughout mitosis. While Aurora kinase A (Aurora A) is localized to the centrosome during interphase, it also accumulates at the spindle pole and mitotic spindle during mitosis.^2^ While Aurora kinase B (Aurora B) is localized to nucleus during interphase, accumulates at chromosomes during prophase, and at inner kinetochores at prometaphase and metaphase, and moves to the central mitotic spindle at anaphase and to the midbody at telophase and cytokinesis.^3^ Aurora C is localized at the centrosome during interphase, at the chromosomes from prophase to metaphase, at the spindle midzone at anaphase, and at the midbody at telophase and cytokinesis.^4^, ^5^ The functions of Aurora kinases are critical for progression of each mitotic step and for completion of cytokinesis. Aurora A controls centrosome maturation, centrosome separation, mitotic entry, chromosome alignment, bipolar spindle formation, and cytokinesis.^6^, ^7^ Aurora B, a component of the chromosomal passenger complex (CPC), which includes INCENP, Survivin, and Borealin, regulates chromosome condensation, chromosome bi‐orientation, the spindle assembly checkpoint (SAC), sister chromatid and centromeric cohesion, spindle assembly/disassembly, and cytokinesis.^8^ Finally, Aurora C regulates division of meiotic and mitotic cells, and compensates for the function of Aurora B in the CPC complex in mitotic cells.^9^, ^10^

Dysregulated expression of Aurora kinases results in accumulation of chromosomal stability.^9^, ^11^ In fact, Aurora kinases are highly overexpressed in diverse cancers.^12^, ^13^, ^14^ Therefore, inhibiting Aurora kinases may be an effective cancer strategy. Numerous Aurora inhibitors have been developed; these include selective Aurora A inhibitors such as alisertib/MLN8237, MK‐5108/VX‐689, MLN8054, and TC‐A 2317; selective Aurora B inhibitors such as barasertib/AZD1152 and hesperadin; and pan‐Aurora inhibitors such as AMG900, AS703569/R‐763, CCT137690, danusertib/PHA‐739358, SNS‐314, VE‐465, and VX‐680/MK‐0457/tozasertib. Pan‐Aurora inhibitors are less well studied than specific Aurora inhibitors.^15^, ^16^

AMG900, a pan‐Aurora inhibitor, inhibits Aurora A/B/C with an IC_50_ of 5, 4, and 1 nmol/L, respectively.^17^, ^18^ However, the mechanism by which AMG900 dysregulates mitotic progression is unknown. AMG900 inhibits proliferation of different cancer cell lines, including adrenal cortical carcinoma,^17^ breast cancer,^18^, ^19^ colon cancer,^20^ medulloblastoma,^21^ and prostate cancer.^22^ AMG900 has been tested against advanced solid tumors and acute myeloid leukemia in Phase 1 clinical trials.^23^, ^24^ However, its effect on glioblastoma has not been studied. Glioblastoma is a very aggressive malignant tumor, and the 5‐year survival rate is <5%.^25^ However, chemotherapeutic options are very limited because few available chemotherapeutic agents can cross the blood‐brain barrier (BBB). Here, we show that AMG900 inhibits proliferation of glioblastoma cells by inducing mitotic catastrophe, suggesting its potential utility as a treatment for glioblastoma.

## 2 MATERIALS AND METHODS

### 2.1 Cell lines

A172, U‐87MG, and U‐118MG glioblastoma cells were obtained from the American Type Culture Collection (Lot number; 62177118, 61978364, and 62996838, respectively). A172 and U‐118MG cells were maintained in Dulbecco's modified Eagle's medium (Welgene Inc., Gyeongsangbuk‐do, Korea; LM001‐05) supplemented with 10% fetal bovine serum, 100 U/mL penicillin G sodium, 100 μg/mL streptomycin sulfate, and 0.25 μg/mL amphotericin B. U‐87MG cells were maintained in Eagle's Minimum Essential Medium (Welgene Inc.; LM007‐07) with the same supplements. Cells were incubated at 37°C in a 5% CO_2_ incubator. The cell lines were routinely tested for the presence of mycoplasma with Hoechst staining.

### 2.2 Drug treatment

Cells were treated with AMG900 (Selleck Chemicals, Houston, TX, USA; S2719) and BI2536 (Selleck Chemicals; S1109) for the indicated times. AMG900 and BI2536 were dissolved in DMSO as a vehicle. The final concentration of vehicle in culture medium was 0.1% (V/V).

### 2.3 Cell viability assay

Cell growth was assessed using the trypan blue exclusion test.^26^ Cells were treated with various concentrations of AMG900. After incubation for the indicated times, cells were detached and mixed with 0.4% trypan blue solution. The number of live cells that did not take up trypan blue was then counted.

### 2.4 Clonogenic assay

A172 cells were treated with DMSO or AMG900 for 24 hours. Then, the cells (3 × 10^3^ cells per 60 mm dish) were plated and then incubated for 14 days. After removal of the medium, cells were rinsed with phosphate‐buffered saline (PBS), fixed in acetic acid:methanol (1:7, vol/vol) at room temperature for 5 minutes and then stained with staining solution (0.5% crystal violet in 25% methanol).^27^ Colonies were counted on triplicate dishes, and independent experiments were repeatedly performed.

### 2.5 Preparation of crude cell extracts and Western blotting

Cells were lysed on ice for 10 minutes using NETN lysis buffer (100 mmol/L NaCl, 1 mmol/L EDTA, 20 mmol/L Tris‐HCl, 0.5% Nonidet P‐40, 50 mmol/L β‐glycerophosphate, 10 mmol/L NaF, and 1 mmol/L Na_3_VO_4_) containing a protease inhibitor cocktail (Millipore, Temecula, CA, USA; 535140). After centrifugation at 12, 000 *g* for 5 minutes, the supernatant was saved as a crude cell extract. This was boiled in Laemmli buffer and loaded onto a SDS‐polyacrylamide gel. Western blotting was performed according to a standard protocol. The following antibodies were used for Western blotting: Cyclin A (Santa Cruz Biotechnology, Dallas, TX, USA; sc‐751), Cyclin B1 (Santa Cruz Biotechnology; sc‐752), Cyclin D1 (Santa Cruz Biotechnology; sc‐753), p21 (Millipore; OP64), p53 (Santa Cruz Biotechnology; sc‐126), PARP‐1 (Santa Cruz Biotechnology; sc‐7150), Aurora A‐pT288 (Cell Signaling, Danvers, MA, USA; 3079), Aurora A (BD Biosciences, San Jose, CA; 610938), Aurora A‐pT288/Aurora B‐pT232/Aurora C‐pT198 (Cell Signaling; 2914), Aurora B (Cell Signaling; 3094), BubR1 (BD Biosciences; 612503), PLK1‐pT210 (Santa Cruz Biotechnology; sc‐135706), PLK1 (Cell Signaling; 4513), β‐actin (Cell Signaling; 4970), and GAPDH (Santa Cruz Biotechnology; sc‐25778). BubR1‐pS670 antibody was obtained from immunized rabbit with specific peptide.

### 2.6 Senescence‐associated β‐galactosidase staining

The cells were washed with PBS, then fixed and stained at pH 6.0 using a senescence β‐galactosidases (SA‐β‐gal) staining kit (Cell Signaling; 9860).^28^ Total 200 cells were randomly selected for counting β‐gal‐positive cells.

### 2.7 Cell cycle analysis

Cells were suspended in PBS, and then, 100% ethanol was added to be the final concentration of 70% ethanol while gently vortexing. The fixed cells were permeabilized with 0.25% Triton X‐100 in PBS on ice for 15 minutes. The cells were incubated with anti‐H3‐pS10 (Millipore; 06‐570) antibody for 2 hours and then incubated with FITC‐conjugated goat anti‐rabbit IgG (Jackson ImmunoResearch Laboratories Inc., West Grove, PA, USA; 111‐095‐144) at room temperature in the dark for 1 hour. Cells were incubated with DNase‐free RNase A at 37°C for 30 minutes and then with propidium iodide (PI) at 37°C in the dark for another 30 minutes. The percentage of cells in each cell cycle phase and H3‐pS10‐positive cells were determined by flow cytometry.

### 2.8 Immunofluorescence staining

Cells were grown on coverslips and treated with indicated drugs. The cells were fixed with 3% paraformaldehyde solution at room temperature for 10 minutes and then permeabilized with 0.5% Triton X‐100 at room temperature for 5 minutes. The cells were incubated with antibody against Aurora A (BD Biosciences; 610938), Aurora B (Santa Cruz Biotechnology; sc‐25426), PLK1 (Santa Cruz Biotechnology; sc‐17783), BubR1 (BD Biosciences; 612503), or CREST (ImmunoVision, Springdale, AR, USA; HCT‐0100) at 37°C for 20 minutes and then incubated with corresponding secondary antibody at 37°C for 20 minutes. For the staining with α‐tubulin (Abcam, Cambridge, United Kingdom; 18251) and pericentrin (Abcam; 28144) antibodies, the cells were fixed with cold methanol at −20°C for 20 minutes and then rehydrated in PBS three times. The cells were postfixed with paraformaldehyde and permeabilized as described above. The nuclei were counterstained with Hoechst 33342. After a final wash with PBS, coverslips were mounted with antifade solution containing para‐phenylenediamine and glycerol in PBS. Stained cells were observed under a laser‐scanning confocal microscope (Carl Zeiss, Oberkochen, Germany; LSM700). One hundred and fifty cells were randomly selected, and the number of cells containing multi‐ and micronuclei and centrosomes was counted in a blinded manner. One hundred cells undergoing mitosis and cytokinesis were randomly selected, and the mitotic phases were counted.

### 2.9 Live‐cell imaging

The TSiN‐H2B‐RFP lentiviral construct was a kind gift from Dr. P. J. Galardy (Mayo Clinic). Lentivirus was prepared by transfecting HEK293T cells with the TSiN‐H2B‐RFP lentiviral plasmid, a psPAX2 packaging plasmid, and a pMD2.G envelope plasmid. A172 cells were infected with lentivirus encoding H2B‐RFP in the presence of 8 μg/mL polybrene. Time‐lapse imaging was then performed using a Cell Observer (Cell Observer Living Cells, Carl Zeiss) equipped with a camera. Frames were recorded every 5 minutes. Cell morphology was visualized under a phase‐contrast microscope, and red fluorescence was detected as described previously.^27^

### 2.10 Data and statistical analysis

All assays were repeated more than three times, and data are expressed as the mean ± standard error of mean (SEM). For the clonogenic assay, the percentage of surviving DMSO‐treated controls cells was set as 100% with no variance (SEM = 0) to reduce inter‐experimental variation. Statistical analysis was performed using SPSS software (IBM, Armonk, NY, USA; version 23). Differences between two groups were evaluated using an unpaired Student's *t* test (parametric analysis) or the Mann‐Whitney *U* test (nonparametric analysis). Differences between three or more groups were evaluated using one‐way analysis of variance (ANOVA) followed by Tukey's honest significant difference (HSD) (parametric analysis) or using the Kruskal‐Wallis test followed by Dunn's multiple comparison test (nonparametric analysis). Post hoc tests were run only if F achieved *P *<* *0.05, and there was no significant inhomogeneity. Statistical differences were considered significant at *P *<* *0.05 and are indicated by *, ^#^, or ^+^ as described in figure legends.

## 3 RESULTS

### 3.1 AMG900‐treated glioblastoma cells show growth defects

**Figure 1:**
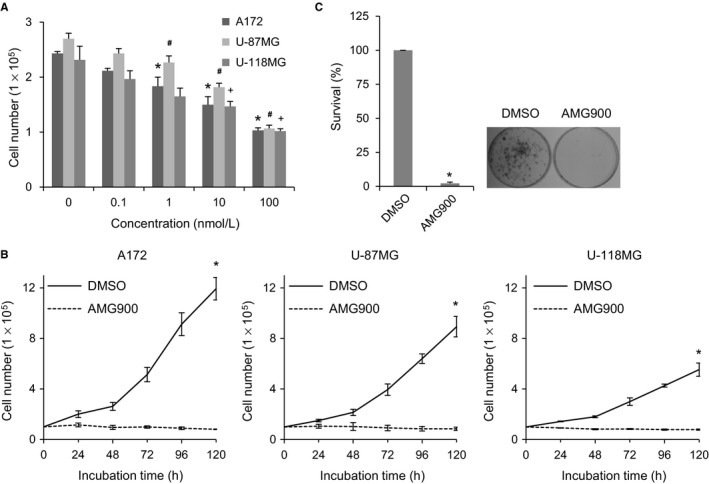
AMG900 reduces proliferation of glioblastoma cells. (A and B), A172, U‐87MG, and U‐118MG cells were treated with AMG900, and viability was determined in a trypan blue exclusion test. A, Cells were treated with various concentrations of AMG900 for 24 h. *P <* *0.05, ^#^ *P *<* *0.05, and ^+^ *P *<* *0.05; significantly different from corresponding DMSO‐treated A172, U‐87MG, and U‐118MG cells, respectively (one‐way ANOVA followed by Tukey's HSD test). (B) Cells were treated with 100 nmol/L AMG900 for the indicated times. *P <* *0.05; significantly different from DMSO‐treated cells (Kruskal‐Wallis test followed by Dunn's test). (C) A172 cells were treated for 24 h with 100 nmol/L AMG900. After washing out AMG900, the cells were seeded and colony growth was examined. Colonies were counted after 14 d. *P <* *0.05; significantly different from DMSO‐treated cells (Mann‐Whitney *U* test).

We examined the effects of AMG900 on the growth of glioblastoma cells. Treatment with AMG900 reduced growth of A172, U‐87MG, and U‐118MG cells in a concentration‐dependent manner (from 0.1 to 100 nmol/L; Figure 1A). In addition, while the number of DMSO‐treated control cells increased in a time‐dependent manner (from 24 to 120 hours), this was not the case for 100 nmol/L AMG900‐treated cells (Figure 1B). To examine the long‐term effects of AMG900, we exposed A172 cells to AMG900 for 24 hours, washed out drug, and examined colony formation after 14 days. AMG900‐treated cells showed significantly lower colony‐forming activity than control cells (Figure 1C), suggesting that short‐term exposure results in irreversible defects in survival. Overall, the data indicate that AMG900 reduces the proliferation of glioblastoma cells.

### 3.2 AMG900‐treated glioblastoma cells undergo senescence

**Figure 2:**
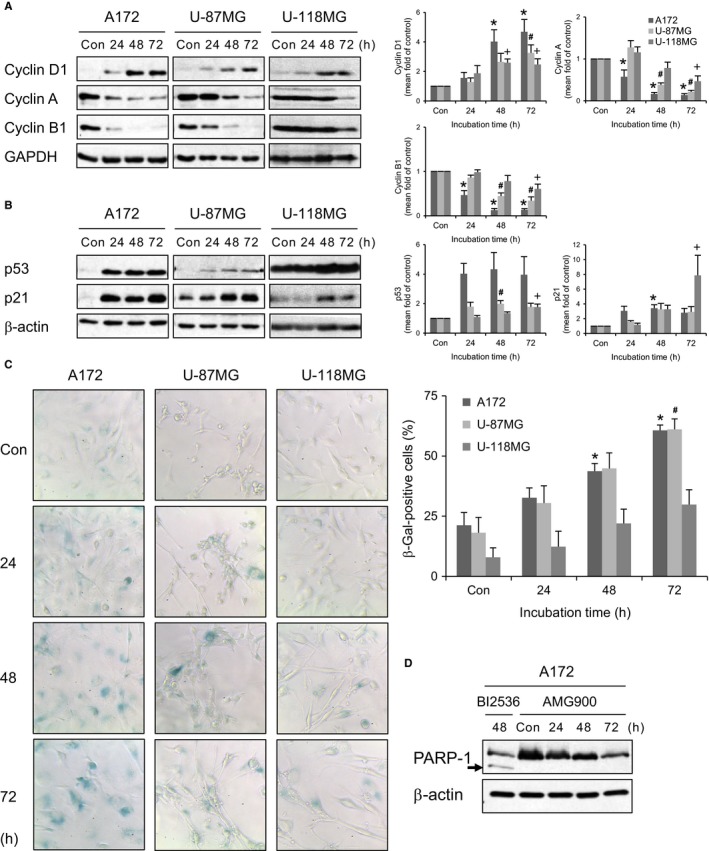
AMG900 induces senescence by upregulating p21. (A‐C) A172, U‐87MG, and U‐118MG cells were treated with 100 nmol/L AMG900 for the indicated times. Con (control) are cells treated with vehicle (DMSO) for 72 h. *P <* *0.05, ^#^ *P *<* *0.05, and ^+^ *P *<* *0.05; significantly different from corresponding control A172, U‐87MG, and U‐118MG cells, respectively (one‐way ANOVA followed by Tukey's HSD test). (A and B) Expression of each protein was evaluated by Western blotting. (C) SA‐β‐gal assay was performed. (D) A172 cells were treated with 50 nmol/L BI2536 or 100 nmol/L AMG900 for the indicated times. Cleaved PARP‐1 indicated by the arrow was evaluated by Western blotting.

AMG900‐induced inhibition of cell proliferation implies cell cycle arrest or cell death. To examine whether AMG900 affects cell cycle arrest, we first measured the level of cyclins D1, A, and B1, the expression of which peaks at G1, G2, and mitosis, respectively. While expression of cyclin D1 increased after AMG900 treatment, that of cyclins A and B1 decreased (Figure 2A). This suggests that cells were arrested in G1. To elucidate the mechanism underlying G1 arrest, we measured the levels of p53 and its transcriptional target gene p21. Both p53 and p21 were upregulated as incubation time increased, although the degree of increase was different between cell lines (Figure 2B). Finally, p21 halted cell cycle progression by inhibiting the CDK/cyclin complex at G1 phase. However, it is unclear whether upregulation of p21 was induced by p53 activation because we do not know whether the p53 mutation harbored by A172 and U‐118MG cells affects its function as a transcription factor (A172, p53 mutant; U‐87MG, p53 wild; U‐118MG, p53 mutant).^29^, ^30^ To further characterize G1 cell cycle arrest in these cells, we stained them for senescence‐associated β‐galactosidase (SA‐β‐gal). Treatment with AMG900 increased the number of β‐gal‐positive cells in all three cell lines in a time‐dependent manner, although the increase in the number of β‐gal‐positive U‐118MG cells was not statistically significant (Figure 2C). Next, to determine whether poor proliferation was due to cell death or autophagy, we examined expression of cleaved PARP‐1 (Figure 2D) and LC3‐II (data not shown), markers for apoptosis and autophagy, respectively; however, we found no changes in A172 cells after treatment with AMG900. As reported in pediatric glioma SF188 cells,^31^ BI2536, a PLK1 inhibitor, induced apoptosis determined by PARP‐1 cleavage in A172 cells (Figure 2D). In addition, the number of apoptotic cells identified by Annexin V/PI staining did not increase in AMG900‐treated A172 cells (data not shown). Therefore, the data indicate that poor survival of AMG900‐treated cells was due mainly to senescence resulting from irreversible G1 arrest.

### 3.3 AMG900‐treated cells accumulate aneuploidy

**Figure 3:**
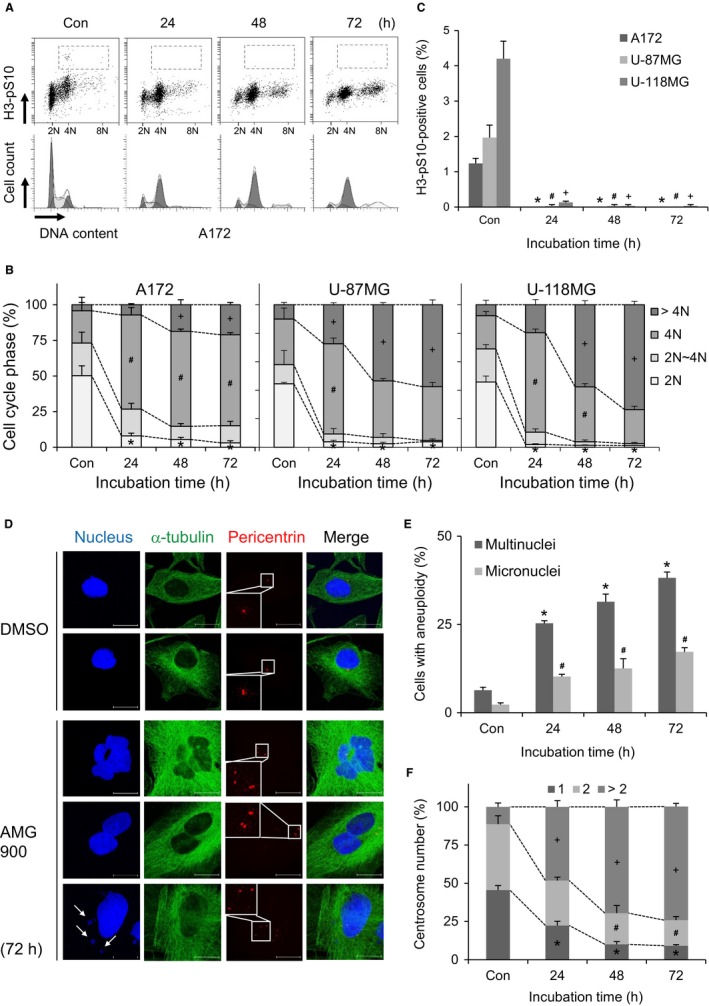
AMG900 causes accumulation of chromosomal instability. (A‐C) A172, U‐87MG, and U‐118MG cells were treated with 100 nmol/L AMG900 for the indicated times. Con (control) cells were treated with vehicle (DMSO) for 72 h. (A‐C) DNA content (B) and proportion of mitotic cells (C) were determined flow cytometry by staining cells with PI and anti‐H3‐pS10 antibody. (B) *P <* *0.05, ^#^ *P *<* *0.05, and ^+^ *P *<* *0.05; significantly different from corresponding 2N, 4N, and >4N of control cells, respectively (one‐way ANOVA followed by Tukey's HSD test). (C) *P <* *0.05, ^#^ *P *<* *0.05, and ^+^ *P *<* *0.05; significantly different from corresponding control A172, U‐87MG, and U‐118MG cells, respectively (one‐way ANOVA followed by Tukey's HSD test). (D‐F) A172 cells were treated with 100 nmol/L AMG900 for the indicated times. Con (control) cells were treated with vehicle (DMSO) for 72 h. (D) Nuclei, cell boundary membrane, and centrosomes were visualized by staining with Hoechst, anti‐α‐tubulin, and anti‐pericentrin antibodies, respectively. Scale bars in panels, 10 μm. Arrows indicate micronuclei. (E) Cells with multinuclei and micronuclei were counted. *P <* *0.05 and ^#^ *P *<* *0.05; significantly different from corresponding control cells (one‐way ANOVA followed by Tukey's HSD test). (F) The number of centrosomes in interphase cells was counted. *P <* *0.05, ^#^ *P *<* *0.05, and ^+^ *P *<* *0.05; significantly different from corresponding control cells with 1, 2, and >2 centrosomes, respectively (one‐way ANOVA followed by Tukey's HSD test).

Next, we asked why AMG900 induces irreversible G1 arrest. To determine whether AMG900‐treated cells undergo abnormal cell division, we examined their cell cycle profile (Figure 3A). First, accumulation of cells with more than 4N DNA content increased significantly following AMG900 treatment (Figure 3A,B), indicating that AMG900‐treated cells exhibit polyploidy. Second, we found that the level of H3‐pS10, a substrate for Aurora A/B/C and a marker of mitosis,^32^ in cells with 4N and 8N DNA content was significantly lower in AMG900‐treated cells than in DMSO‐treated control cells (Figure 3A,C). It implicates that the polyploid cells were not proliferating. However, because H3‐S10 is phosphorylated by Aurora kinases, the low level of H3‐pS10 might just represent low activity of Aurora kinase. Overall, the data suggest that senescence induced by AMG900 is due to induction of polyploidy. Indeed, there was a significant and time‐dependent increase in the number of cells within the AMG900‐treated A172 cell population that harbored either a multi‐lobular nucleus or multiple nuclei (Figure 3D,E). In addition, A172 cells harbored micronuclei after exposure to AMG900 (Figure 3D,E). Aneuploidy, including micronucleation and multinucleation, probably results from chromosomal missegregation and a failure of cytokinesis. In addition, cells with an abnormal number of centrosomes indicated by staining for pericentrin, a component of the pericentriolar material (PCM), increased significantly over AMG900 treatment time (Figure 3D,F). While DMSO‐treated A172 cells normally contained one or two centrosomes, AMG900‐treated A172 cells contained multiple centrosomes. This suggests that AMG900‐treated cells contain supernumerary centrosomes following a failure of cytokinesis. In addition, AMG900‐treated U‐87MG and U‐118MG cells showed multinucleation, micronucleation, and multiple centrosomes (data not shown). Taken together, the results suggest that chromosomal missegregation and a failure of cytokinesis result in aneuploidy.

### 3.4 AMG900‐treated cells undergo abnormal mitotic progression

**Figure 4:**
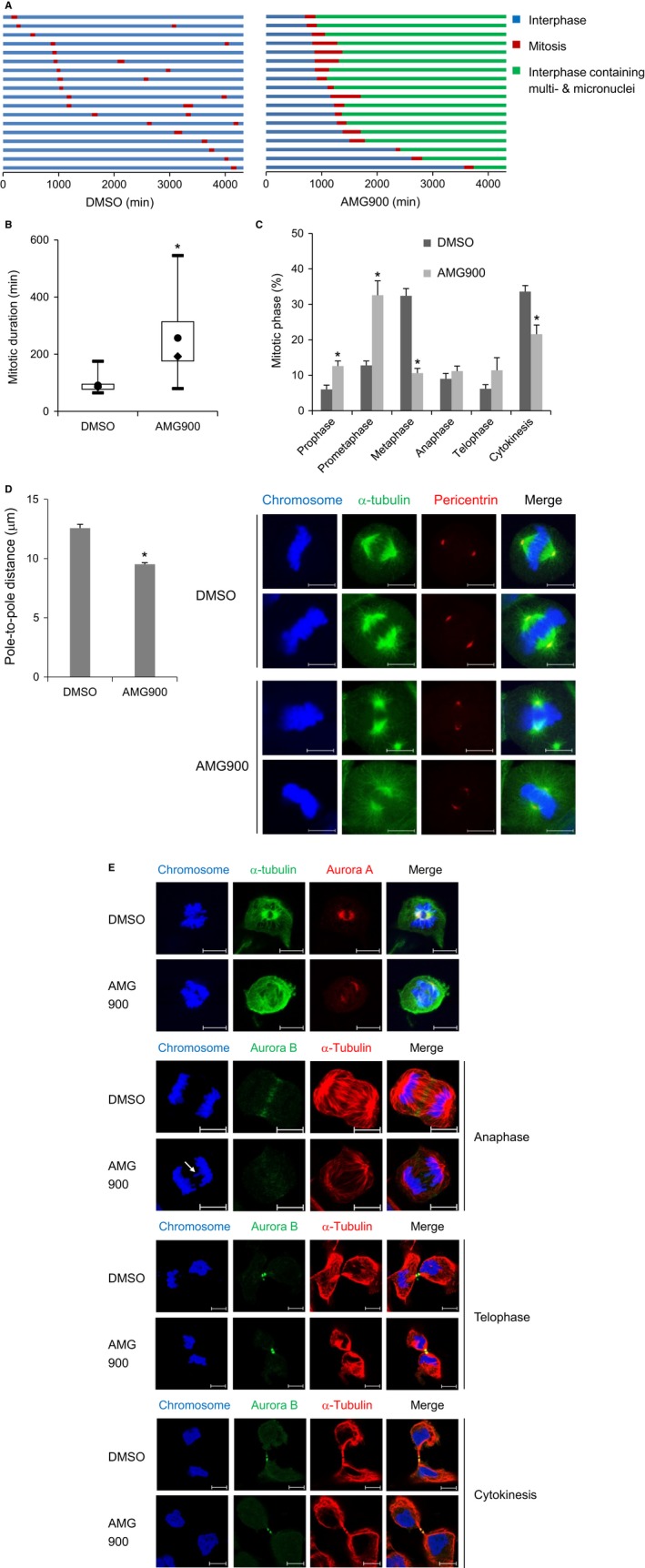
AMG900 causes prolonged mitotic progression due to a defect in mitotic spindle formation. (A and B) A172 cells were treated with 100 nmol/L AMG900 and then subjected to live‐imaging fluorescence microscopy to track individual cells for 72 h (=4320 min). (A) The color of the bar denotes the status of the cells: normal interphase, mitosis, and interphase containing aneuploidy. The length of the bar denotes the duration of each status. Each horizontal bar represents one cell (n = 18). (B) The duration of mitosis is represented as a box‐and‐whisker plot. Boxes, interquartile range; whiskers, minimum, and maximum values; circles, average values; diamonds, median values. *P <* *0.05; significantly different from DMSO‐treated cells (Mann‐Whitney *U* test). (C‐E) A172 cells were treated with 100 nmol/L AMG900 for 30 min. (C) The number of each mitotic phase was counted. *P <* *0.05; significantly different from corresponding DMSO‐treated cells (Mann‐Whitney *U* test). (D) The pole‐to‐pole distance was measured as the length between mitotic spindle poles stained with an anti‐pericentrin antibody. *P <* *0.05; significantly different from DMSO‐treated cells (unpaired Student's *t* test). (E) Localization of Aurora A and Aurora B was determined by staining with the indicated antibodies. Scale bars in panels, 10 μm. Arrows indicate lagging chromosomes.

Accumulation of aneuploidy implies that AMG900 induces abnormal mitotic division. Therefore, to monitor progression of mitosis, we performed live‐cell imaging and observed the mitotic phases. While the majority of DMSO‐treated A172 cells underwent two rounds of cell division over 72 hours, AMG900‐treated A172 cells exiting mitosis remained at interphase; these cells contained multinuclei and micronuclei (Figure 4A). Assuming that AMG900‐treated cells had 8N DNA content (Figure 3A), the cells underwent a further round of DNA replication without a second mitosis, a process referred to as endoreduplication. In addition, the duration of mitosis was longer in AMG900‐treated cells than in DMSO‐treated cells (Figure 4B). Overall, the data indicate that AMG900 causes cell cycle arrest following abnormal mitotic delay. Next, to check which mitotic step is defective, we counted the number of cells at each mitotic phase by examining alignment of condensed mitotic chromosomes, breakdown and formation of the nuclear envelope, and cytoplasmic division after 30‐min exposure to AMG900. The number of prometaphase step in AMG900‐treated cells increased significantly, but the number of metaphase step fell markedly (Figure 4C). This suggests that there is a hurdle that cells must pass over to move from prometaphase to metaphase. To investigate why there is a delay in this transition, we checked the positioning of mitotic spindle poles by examining PCM localization as indicated by pericentrin staining. The pole‐to‐pole distance in AMG900‐treated cells was significantly shorter than that in DMSO‐treated control cells (Figure 4D). In addition, assembly of mitotic spindles in AMG900‐treated cells was clearly defective (Figure 4D). The absence of a pulling force exerted by microtubules might result in wider alignment of condensed chromosomes. Therefore, cells delay the transition from prometaphase to metaphase. Overall, the data suggest that AMG900 leads to a defect in mitotic spindle assembly, resulting in chromosomal missegregation. Errors in chromosomal segregation produce lagging chromosomes. In fact, lagging chromosomes which form a micronucleus after cell division were detected in anaphase following a 30‐min treatment with AMG900 (Figure 4E). In addition, we examined localization of Aurora A to the spindle pole and mitotic spindle. Aurora A localized at the spindle pole in both control‐ and AMG900‐treated cells (Figure 4E), indicating that Aurora kinases activity is not necessary for recruitment of Aurora A to the spindle pole. However, because mitotic spindle assembly was defective in AMG900‐treated cells, we did not observe clear localization of Aurora A at the mitotic spindle (Figure 4E). Next, we examined the spatiotemporal dynamics of Aurora B, a component of the chromosomal passenger complex (CPC), from anaphase to cytokinesis. A defect in Aurora B function results in errors in chromosome segregation, spindle elongation, and cytokinesis.^33^, ^34^ The enrichment of Aurora B at central spindle at anaphase was defective in AMG900‐treated cells, while Aurora B localized to cleavage furrow at telophase and midbody at cytokinesis in both control‐ and AMG900‐treated cells (Figure 4E). It suggests that the defective recruitment of CPC to central spindle dysregulates chromosomal segregation, and the following production of lagging chromosomes prevents abscission due to inactivation of abscission checkpoint in the presence of Aurora kinase inhibitor.^35^, ^36^ Overall, AMG900 causes chromosomal missegregation and a failure of cytokinesis.

### 3.5 AMG900‐treated cells override the SAC

**Figure 5:**
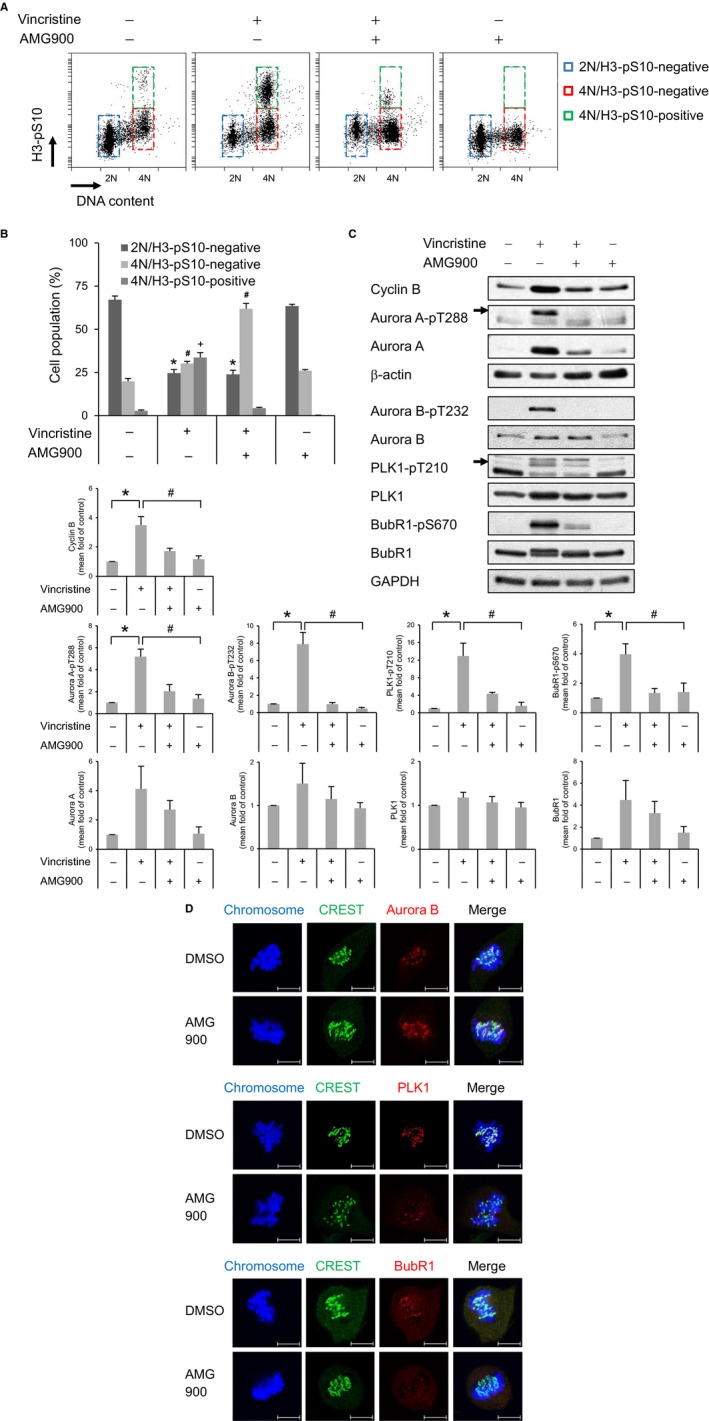
AMG900 inactivates the spindle assembly checkpoint. (A‐C) A172 cells were pretreated with 10 nmol/L vincristine for 16 h, followed by 100 nmol/L AMG900 for 2 h. DNA content (A) and proportion of mitotic cells (B) were determined flow cytometry after staining cells with propidium iodide (PI) and an anti‐H3‐pS10 antibody. *P <* *0.05, ^#^ *P *<* *0.05, and ^+^ *P *<* *0.05; significantly different from the corresponding populations of control cells (one‐way ANOVA followed by Tukey's HSD test). (C) Expression of each protein was determined by Western blotting. *P <* *0.05, and ^#^ *P *<* *0.05; significantly different from control cells and vincristine‐treated cells, respectively (one‐way ANOVA followed by Tukey's HSD test). (D) Localization of Aurora B, PLK1, and BubR1 in A172 cells treated with 100 nmol/L AMG900 for 30 min was determined by staining with the indicated antibodies. Scale bars in panels, 10 μm.

Next, we asked how AMG900‐treated cells exit mitosis in the presence of detrimental mitotic defects. To enrich mitosis‐arrested cells, A172 cells were treated with vincristine, a microtubule destabilizer, which activates the SAC. Treatment with vincristine alone induced cell cycle arrest of the 4N/H3‐pS10‐positive population, but treatment with AMG900 led to a significant reduction in the number of 4N/H3‐pS10‐positive cells (Figure 5A,B). This indicates that AMG900 inhibits the phosphorylation of H3‐S10 by Aurora kinases and possibly inactivates the SAC. Failure of the SAC was verified by examining activation of SAC‐related proteins such as phosphorylation on Aurora A‐T288, Aurora B‐T232, PLK1‐T210, and BubR1‐S670, which is mediated by Aurora A, Aurora B, Aurora A, and Mps1, respectively. We found that vincristine‐mediated increases in expression of phospho‐Aurora A‐T288, Aurora B‐T232, PLK1‐T210, and BubR1‐S670 decreased after treatment with AMG900 (Figure 5C; lane 2 vs lane 3), indicating that AMG900 inactivates vincristine‐induced SAC. In addition, the level of total cyclin B, a substrate for the anaphase‐promoting complex/cyclosome (APC/C) E3 ubiquitin ligase complex, was downregulated in cells treated with vincristine followed by AMG900 (Figure 5C; lane 2 vs lane 3). This suggests that SAC inactivation results in APC/C‐mediated degradation of the substrate and onset of anaphase. The above results were obtained under conditions of drug‐induced SAC activation. Next, to investigate whether AMG900 induces a defect in mitotic progression in the absence of drug‐mediated SAC activation, we treated cells with AMG900 for 30 minutes and then checked recruitment of SAC‐related proteins to the kinetochore of prometaphase chromosomes, as determined by staining with CREST, an anti‐centromere autoantibody derived from serum of patients with CREST syndrome (limited scleroderma). As expected, Aurora B, PLK1, and BubR1 were recruited to the unattached kinetochore in control cells. By contrast, while Aurora B was still recruited to the unattached kinetochore in AMG900‐treated cells, recruitment of PLK1 was partially defective and recruitment of BubR1 was completely abolished (Figure 5D). These data demonstrate that activity of Aurora kinases is not required for recruitment of Aurora B, but is, at least in part, necessary for recruitment of PLK1 and BubR1 to unattached kinetochores. Overall, the data suggest that inactivation of Aurora kinases results in incomplete activation of the SAC. Inactivation of the SAC allows onset of anaphase without correcting errors, thereby inducing aneuploidy.

## 4 DISCUSSION

Here, we showed that AMG900, a pan‐Aurora inhibitor, inhibits proliferation of glioblastoma cells by inducing cell cycle arrest. Dysfunction of mitotic progression results in accumulation of chromosomal instability, ultimately leading to senescence. Therefore, AMG900 may be a potential treatment for glioblastoma.

Aurora kinases are thought to be a potential therapeutic target for glioblastoma. Expression of mRNA encoding Aurora A and B is higher in glioma than in normal brain tissue.^37^, ^38^, ^39^, ^40^ In addition, higher expression of Aurora A mRNA or protein is associated with poor survival of glioblastoma patients.^40^, ^41^, ^42^ Expression of Aurora B protein is more common in patients with short survival times than in those with long survival times.^43^ Therefore, several trials have attempted to use an Aurora kinase inhibitor to treat glioblastoma. Specific Aurora A inhibitors (e.g., alisertib^44^, ^45^ MLN8237^39^) or an Aurora B inhibitor (AZD1152‐HQPA^46^) improved survival rates of mice bearing glioblastoma xenografts.^39^, ^44^, ^45^, ^46^

Genetic depletion or pharmacological inhibition of Aurora A and B results in aberrant cell division.^11^ Suppression of Aurora A induces formation of monopolar spindles and aneuploidy, including multinucleation and micronucleation.^47^, ^48^, ^49^ Suppression of Aurora B induces formation of multipolar spindles and failure of cytokinesis, leading to multinucleation.^50^, ^51^, ^52^ Suppression of both Aurora A and B results in formation of multipolar spindles and mitotic exit without chromosomal segregation, resulting in polyploidy.^53^ Although no specific inhibitor of Aurora C has been developed, depleting Aurora C and overexpression of kinase‐dead Aurora C in mitotic cells results in multinucleation.^54^, ^55^ The outcome of pan‐Aurora inhibitor treatment reflects suppression of Aurora A, B, and C. Treatment with VX‐680/MK‐0457/tozasertib, the most well studied pan‐Aurora inhibitor, induces formation of monopolar spindles^56^, ^57^, ^58^ or multipolar spindles,^59^, ^60^ ultimately leading to polyploidy. Mitotic duration in VX‐680‐treated cells is prolonged.^56^, ^57^ Both Danusertib/PHA‐739358^61^, ^62^, ^63^, ^64^, ^65^, ^66^ and AMG900^17^, ^18^, ^19^, ^20^, ^22^ induce polyploidy; however, the mechanism by which pan‐Aurora inhibitors disrupt mitotic progression and induce polyploidy is unclear. Previous reports just demonstrate that AMG900 induces formation of polyploid cells with low level of H3‐pS10 in liposarcoma cells,^67^ breast cancer cells,^18^, ^19^ prostate cancer cells,^22^ and colon cancer cells.^20^ AMG900‐treated colon cancer cells entering mitosis abort cell division without proper chromosome congression and alignment.^20^ Here, we show that AMG900 causes accumulated chromosomal instability by defects in mitotic spindle formation, SAC activation, chromosome segregation, and cytokinesis.

Accumulation of chromosomal instability induced by pan‐Aurora inhibitors ultimately inhibits proliferation of several cancer cell lines by inducing apoptosis.^17^, ^18^, ^19^, ^20^, ^21^, ^22^, ^66^, ^68^, ^69^ Apoptotic cell death does not require functional p53. VX‐680‐induced apoptosis and endoreduplication readily increases in p53‐dysfunctional cells than in p53 wild‐type A549 or HCT116 cells.^70^, ^71^ The sensitivity of breast cancer cells to AMG900 also correlates with p53 loss‐of‐function mutations and low expression of p21.^18^ This suggests that pan‐Aurora inhibitor‐induced apoptosis is mediated via a p53‐independent pathway. However, we show here that AMG900 does not induce apoptosis in glioblastoma cells. This discrepancy might be due to different cancer cell types. Reduced cancer cell growth is also attributed to senescence. AMG900 induces senescence in prostate cancer cells by upregulating p21.^22^ We also found that AMG900‐treated glioblastoma cells underwent senescence, with upregulated expression of p21 irrespective of whether they harbored a p53 mutation. Further studies should examine whether only p21 is responsible for AMG900‐induced senescence.

In vitro studies suggest that AMG900 is an effective inhibitor of various cancers, including breast, colon, and prostate cancer, adrenal cortical carcinoma, and medulloblastoma.^17^, ^18^, ^19^, ^20^, ^21^, ^22^ In vivo tumor xenografts of breast, colon, and prostate cancer cells also demonstrate that AMG900 is a potential anti‐cancer drug.^19^, ^20^, ^22^ In addition, AMG900 acts synergistically with other anti‐cancer drugs^17^ and histone deacetylase inhibitors^21^, ^22^ to sensitize cells to apoptosis. AMG900 also inhibits proliferation of paclitaxel‐resistant breast and lung cancer cells.^19^, ^20^ Therefore, AMG900 may be a viable therapeutic option for diverse cancer types.

Glioblastoma is a tumor that is difficult to treat due to the limited range of available chemotherapeutic agents. Therefore, development of drugs that are small, lipid soluble molecules that can cross the BBB by transmembrane diffusion is required.^72^ AMG900 could be such a drug if a delivery system that can penetrate or bypass the BBB is developed.

## CONFLICT OF INTEREST

We declare that no competing of interest and conflicts of ethics involved in the manuscript.
